# Impact of phased COVID-19 vaccine rollout on anxiety and depression among US adult population, January 2019–February 2023: a population-based interrupted time series analysis

**DOI:** 10.1016/j.lana.2024.100852

**Published:** 2024-08-09

**Authors:** Yusen Zhai, Mengchen Fan, Baocheng Geng, Xue Du, Scott Snyder, Larrell Wilkinson

**Affiliations:** a Department of Human Studies, The University of Alabama at Birmingham, Birmingham, AL, USA; b Department of Computer Science, The University of Alabama at Birmingham, Birmingham, AL, USA; c Heersink School of Medicine, The University of Alabama at Birmingham, Birmingham, AL, USA

**Keywords:** Health policy, Anxiety, Depression, Vaccines, COVID-19, Deep learning

## Abstract

**Background:**

Existing research lacks information on the potential impacts of multi-phased coronavirus disease 2019 (COVID-19) vaccine rollouts on population mental health. This study aims to evaluate the impact of various COVID-19 vaccine rollout phases on trends and prevalence of anxiety and depression among US adults at a population level.

**Methods:**

We performed a US population-based multi-intervention interrupted time series analysis through Deep Learning and autoregressive integrated moving average (ARIMA) approaches, analyzing 4 waves of US CDC's Behavioral Risk Factor Surveillance System (BRFSS) data (January 2019–February 2023) to assess changes in the weekly prevalence of anxiety and depression following interruptions, including all major COVID-19 vaccine rollout phases from 2020 to early 2023 while considering pandemic-related events.

**Findings:**

Among 1,615,643 US adults (1,011,300 [76.4%] aged 18–64 years, 867,826 [51.2%] female, 126,594 [16.9%] Hispanic, 120,380 [11.9%] non-Hispanic Black, 1,191,668 [61.7%] non-Hispanic White, and 113,461 [9.5%] other non-Hispanic people of color), we found that three COVID-19 vaccine rollout phases (ie, prioritization for educational/childcare workers, boosters for all US adults, authorization for young children) were associated with a 0.93 percentage-point (95% CI −1.81 to −0.04, p = 0.041), 1.28 percentage-point (95% CI −2.32 to −0.24, p = 0.017), and 0.89 percentage-point (95% CI −1.56 to −0.22, p = 0.010) reduction, respectively, in anxiety and depression prevalence among the general US adult population despite an upward trend in the prevalence of anxiety and depression from 2019 to early 2023. Among different population groups, Phase 1 was associated with increases in anxiety and depression prevalence among Black/African Americans (2.26 percentage-point, 95% CI 0.24–4.28, p = 0.029), other non-Hispanic people of color (2.68 percentage-point, 95% CI 0.36–5.00, p = 0.024), and lower-income individuals (3.95 percentage-point, 95% CI 2.20–5.71, p < 0.0001).

**Interpretation:**

Our findings suggest disparate effects of phased COVID-19 vaccine rollout on mental health across US populations, underlining the need for careful planning in future strategies for phased disease prevention and interventions.

**Funding:**

None.

**Research in context**

**Evidence before this study**

Existing research lacks information on the potential impacts of multi-phased COVID-19 vaccine rollouts on population mental health, despite a burgeoning body of literature documenting the changes in the prevalence of mental health conditions among different populations from the pre-pandemic to the peri-pandemic period. We conducted electronic database searches in PubMed to identify published research documenting the prevalence of mental health issues before and during the COVID-19 pandemic without any language restrictions. Our search used the terms (“COVID-19 vaccine” OR “vaccine rollout” OR “vaccine distribution”) AND (“mental health” OR “anxiety” OR “depression”) from inception until January 11th, 2024. Our search was not limited to any particular geographic region or population, considering the global reach of the psychological impacts of the COVID-19 pandemic and the ensuing vaccine rollout. Existing evidence highlights increased anxiety and depression prevalence among the US population after the COVID-19 outbreak. However, there is a noticeable lack of empirical evidence assessing the potential impacts of different phases of the COVID-19 vaccine rollout on mental health.

**Added value of this study**

To the best of our knowledge, this is the first study providing a comprehensive understanding of the disparate effects of phased COVID-19 vaccine rollout on mental health outcomes among US adults. Based on Deep Learning and ARIMA models, this multi-intervention interrupted time series analysis, utilizing four waves of population-level data (*N* = 1,615,643, January 2019–February 2023) from the CDC's BRFSS, addresses the research gap by assessing the changes in the prevalence of anxiety and depression following different phases of the COVID-19 vaccine rollout among the general US adult population and subpopulations. This study provides empirical evidence on the potential psychological impact of phased COVID-19 vaccine rollout, suggesting that three COVID-19 vaccine rollout phases (ie, prioritization for educational/childcare workers, boosters for all US adults, authorization for young children) might have reduced the mental health burden among US adults. Moreover, we found associations of Phase 1 vaccine rollout with significant increases in the prevalence of anxiety and depression among Black/African Americans, other non-Hispanic people of color, and lower-income individuals.

**Implications of all the available evidence**

The empirical evidence gleaned from this study underscores the pressing need to address the disparate effects of the phased vaccine rollout on mental health outcomes across diverse populations. Policymakers and healthcare providers should consider the mental health outcomes tied to the phased vaccine rollout, particularly among vulnerable and marginalized groups. Comprehending the potential mental health implications of the multi-phased vaccine rollout will facilitate the formulation of effective disease prevention and intervention strategies while also mitigating potential psychological impacts. As such, targeted mental health care should be integrated into public health strategies to manage ongoing and future public health crises. Furthermore, this study emphasizes the need for equitable access to vaccines, strategies to confront racism and discrimination in healthcare settings, and efforts to combat vaccine misinformation.

## Introduction

The coronavirus disease 2019 (COVID-19) pandemic intensified pre-existing challenges for many US individuals and families and exacerbated health disparities.^1^ A growing body of literature has documented the multifaceted impacts of the pandemic on mental health, reflecting the socioeconomic and public health upheaval triggered by COVID-19 across diverse US populations.^1^, ^2^, ^3^ Research showed that many US individuals faced compounded stressors and trauma due to high COVID-19-related mortality and morbidity, resulting in an escalation in anxiety and depression levels during the initial stages of the pandemic.^4^, ^5^, ^6^ Following the COVID-19 outbreak, there was a marked increase in the prevalence of anxiety (25.5%) and depression (24.3%), approximately three and four times the rates reported in the equivalent period of 2019 (8.1% and 6.5% respectively),^5^ which could lead to severe public health and economic burdens. The initiation of the COVID-19 vaccine rollout, which commenced in the United States on December 11, 2020, was anticipated to provide many US individuals with a sense of optimism for a return to normalcy. However, the impact of that rollout and subsequent rollout phases on mental health outcomes in the general public remains unclear.

In responding to the escalating physical and mental health crisis associated with COVID-19, government and public health officials collaborated with pharmaceutical companies to allocate resources for the development of COVID-19 vaccines, a crucial factor in controlling COVID-19 transmission and mortality prior to the emergence of highly transmissible variants such as B.1.617.2 (Delta).^7^ Apart from the direct health benefits of the vaccine rollout in reducing COVID-19-related morbidity and mortality,^8^ there is a burgeoning body of research examining the psychological implications of COVID-19 vaccination.^9^, ^10^, ^11^, ^12^ While most studies report reduced adverse mental health symptoms post-vaccination,^9^, ^10^, ^11^, ^12^ few have noted instances of specific psychiatric symptoms.^13^^,^^14^ Research has also suggested that mental health issues are associated with vaccine hesitancy and trust in government and public health officials. The rapid development and emergency use authorization (EUA) of COVID-19 vaccines might have contributed to mistrust in government officials and health systems and misinformation about these vaccines, exacerbating mental health issues and vaccine hesitancy, particularly among vulnerable populations.^15^^,^^16^ Additionally, people with mental disorders are more vulnerable to infectious diseases and misinformation about preventive measures such as vaccines; thus, they may have lower trust in government and public health officials, which can aggravate mental health symptoms and worsen vaccine hesitancy and uptake.^17^ Inequitable COVID-19 vaccine distributions might further erode public trust and mental health.^18^ Moreover, research has suggested that vaccine hesitancy predicts vaccine side effects, indicating the need to address mental health issues (eg, anxiety) associated with vaccine hesitancy to reduce nocebo side effects.^19^^,^^20^ Despite these findings, there remains a significant evidence gap with major public health implications regarding the potential impact of a phased COVID-19 vaccine rollout on mental health among US adults.

The vaccine rollout has been divided into several phases based on priority groups and risk factors (time points 3–6 and 8–11 in Table 1), as outlined by US government officials.^21^^,^^22^ The public health guidelines on distributing the vaccine have likely influenced mental health outcomes in several ways. The availability of the vaccine might offer hope and relief to those affected by the pandemic, potentially enhancing a sense of control and optimism.^23^^,^^24^ Conversely, the phased distribution of the vaccine could instigate stress due to concerns about its accessibility, and the unequal distribution may exacerbate social and health inequalities, potentially amplifying feelings of uncertainty, frustration, and resentment.^25^^,^^26^ Because of the potential mixed psychological effect of phased COVID-19 vaccine rollout, we hypothesized that COVID-19 vaccine rollout phases would be associated with a change in the prevalence of anxiety and depression among US adults while controlling for the potential psychological effects associated with major pandemic-related events (time points 1, 2, and 7 in Table 1).

**Table 1:** Major phases of COVID-19 vaccine rollout and pandemic-related events in the United States.^19^^,^^20^

Interruption variable	Type	Date	Description^19^^,^^20^
1	Pandemic-related event	January 10, 2020	The findings of a novel virus that caused severe unknown pneumonia (later known as COVID-19), first defined as SARS-CoV-2, were published by the CDC and made available to the public.
2	Pandemic-related event	March 13, 2020	The Trump Administration declared a nationwide emergency in light of the public health threat due to COVID-19.
3	Vaccine rollout	December 11, 2020	Phase 1: healthcare workers and certain at-risk groups were eligible for COVID-19 vaccines.
4	Vaccine rollout	March 2, 2021	The US Department of Health and Human Services (DHHS) directed all education/childcare workers to be eligible for COVID-19 vaccines, which led to a significant increase in vaccination coverage/rates.
5	Vaccine rollout	April 19, 2021	Phase 2: all US individuals aged 16+ were eligible for COVID-19 vaccines.
6	Vaccine rollout	May 10, 2021	Adolescents aged 12–15 were eligible for COVID-19 vaccines.
7	Pandemic-related event	July 30, 2021	CDC released findings that showed an increase in breakthrough infections of COVID-19, suggesting concerns over the Delta variant.
8	Vaccine rollout	September 22, 2021	The first COVID-19 booster dose of vaccines was available to certain at-risk US populations.
9	Vaccine rollout	October 29, 2021	Children aged 5–11 were eligible for COVID-19 vaccines.
10	Vaccine rollout	November 21, 2021	All US adults were eligible for a booster dose of vaccines.
11	Vaccine rollout	June 18, 2022	Children aged 6 months to 5 years old were eligible for COVID-19 vaccines.

Early studies^5^^,^^6^ that compared mental health among US individuals between peri-pandemic online surveys with meager response rates and pre-pandemic government benchmark surveys can potentially introduce considerable bias.^27^ Recent research utilizing population-level data suggests that the rise in anxiety and depression is less substantial than early reports indicated.^27^ Despite discrepancies in the magnitude of these results, these studies all employed straightforward pre-/post-comparisons to examine changes in the prevalence of mental health outcomes before and during the pandemic, which is limited in their ability to account for pre-existing underlying short/long-term trends in mental health outcomes and for types of pandemic-related effects. Therefore, this study utilized a multi-invention interrupted time series design as a quasi-experimental approach through the autoregressive integrated moving average (ARIMA) modeling and Deep Learning to address these limitations. Taken together, the aim of this study was to evaluate the association of specific phases of COVID-19 vaccine rollout with the prevalence of anxiety and depression among US adults at a population level.

## Methods

We analyzed secondary, de-identified data from the public source (in our case, CDC's Behavioral Risk Factor Surveillance System, BRFSS^28^); thus, in accordance with the US Department of Health and Human Services (45 CFR §46), the current study does not fall under the category of human participant research and does not require institutional review board review. The study followed the STROBE reporting guideline.

### Sample

BRFSS is the largest continuing health survey system in the world and is an ongoing collaborative project between the US CDC, all US states, and certain US territories.^28^ As the nation's premier health surveillance system, BRFSS conducts monthly both landline and cellular telephone-based surveys to collect data on risk behaviors, chronic health conditions, healthcare accessibility, and the use of preventive services among noninstitutionalized US adults. BRFSS employs a sophisticated weighting methodology named iterative proportional fitting/raking to reduce the potential for bias and ensure sample representativeness, making it an extensive, nationally representative data source to investigate health-related issues on a populational level. Complex survey design and weights are used to reduce nonresponse/selection bias and non-coverage errors. In SPSS, we performed the complex samples function to compute weekly prevalence of clinically significant anxiety and depression from January 2019 to February 2023, enabling the estimates from sample data to represent the entire US adult population in each survey wave.^29^ The median state-level response rate was 49.4% in 2019, 47.9% in 2020, 44.0% in 2021, and 45.1% in 2022–February 2023. This study concentrated on adults from the 50 states and the District of Columbia, with a final sample of 1,615,643 participants (386,448 in 2019, 376,297 in 2020, 401,979 in 2021, 426,923 in 2022, and 23,996 in January–February 2023).

### Measurement

The primary outcome of this study is the prevalence of anxiety and depression. While the core module of BRFSS does not incorporate scales for screening anxiety or depression, we employed a core BRFSS question that shows good concordance with the dichotomous version of the Patient Health Questionnaire-4 (PHQ-4) screening anxiety and depression, which is the same instrument used to measure anxiety and depression in the Household Pulse Survey (HPS) and several other populational online surveys assessing the impact of the pandemic.^27^ Research utilizing the 2018 BRFSS, which has an additional module including the PHQ-4, suggests that clinical levels of anxiety and depression are warranted when the total score of PHQ-4 is greater than or equal to 6.^27^ The core BRFSS question (“Now thinking about your mental health, which includes stress, depression, and problems with emotions, for how many days during the past 30 days was your mental health not good?”) has demonstrated strong diagnostic accuracy and alignment with the PHQ-4 at a cutoff of 6 (with an area under the receiver operating characteristic curve of 0.84).^27^ Hence, aligning with Kessler et al.,^27^ we classified responses to this core BRFSS item into two categories: 15–30 days and 0–14 days. This categorization was chosen as it optimally balanced false positives and false negatives for identifying a PHQ-4 score of 6 or higher (7.2% vs 6.2%), and we utilized this binary variable for our analysis to estimate the weekly prevalence of anxiety and depression. Specifically, this binary variable was coded 1 when participants responded with 15 or more days to this core BRFSS question.^27^

### Statistical analysis

#### ARIMA

To examine the association of specific COVID-19 vaccine rollout phases with trends and prevalence of anxiety and depression, we employed a multi-intervention interrupted time series analysis utilizing ARIMA modeling approach.^30^ This approach generates time-series regression models that account for trends and seasonal variation while also considering potential confounding effects of social processes that may influence an outcome series. Specifically, the ARIMA approach allows researchers to distinguish potential confounding effects of social processes from those associated with the intervention (in this study, COVID-19 vaccine rollout phases) by applying the appropriate seasonal/non-seasonal autoregressive, differencing (integration), and moving average parameters. The moving average component can serve as a smoothing function to reduce noise and account for short-term fluctuations in the data, which could help mitigate the impact of unmeasured or unobserved confounders.^31^^,^^32^ Because of the importance of a stationary time series in ARIMA, we plotted the time series (Supplementary Figure S1) to examine potential trends and seasonality, which can make the time series non-stationary. If the time series are not stationary, differencing can help remove changes in the level of the time series in order to stabilize the mean of a time series, thereby eliminating or reducing trends and seasonality.^33^^,^^34^

The BRFSS data from 2019 to February 2023 were transformed into weekly prevalence of anxiety and depression for the total population and subpopulations stratified by age, race/ethnicity, biological sex, household income, physical health, and whether they were raising children in the household (Table 2). This data was then analyzed on weekly aggregations from January 2019 to February 2023 via ARIMA procedures. Table 1 illustrates the timing of major COVID-19 vaccine rollout phases and pandemic-related events that constituted the interruptions (known as interventions in the quasi-experimental design). In this interrupted time series analysis using a multi-interruption approach, the accurate delineation of intervention variables is crucial for precisely estimating the effects of phased vaccine rollout on anxiety and depression among US adults. Given that the outcome variable is based on assessments of mental health conditions over the preceding 30 days, it is essential to afford participants adequate time to manifest responses to the interruptions included in this time series from January 2019 to February 2023. Hence, to evaluate the impact of an event as an interruption on mental health conditions, we utilize the psychological responses of participants within a 1-month window subsequent to the event. Specifically, in ARIMA models, we assigned the intervention binary variable to begin 1 month (ie, 4 weeks) after the actual date of interventions to estimate participants’ psychological responses to the interruptions. This approach ensures that the temporal dynamics of mental health responses are appropriately captured, aligning the assessment period with the temporal lag inherent in the manifestation of psychological effects post-event. In ARIMA, we computed dummy variables to represent intervention components, where a value of 1 signified the initiation and duration of specific vaccine rollout policies. In the present study, the intervention variable for Phase 1 of the vaccine rollout extended to the point where educational and childcare workers were prioritized for vaccines. Subsequently, the intervention variable for this prioritization persisted until Phase 2 commenced, at which juncture all US individuals aged 16 and older became eligible for vaccination.

**Table 2:** Demographic characteristics of US adult population by year.

Characteristic	Yearly cohort, No. (%)^a^				
	2019 cohort (n = 386,448)	2020 cohort (n = 376, 297)	2021 cohort (n = 401, 979)	2022 cohort (n = 426,923)	Jan–Feb 2023 cohort (n = 23,996)
Age group, y					
18–64	238,814 (77.4)	239,469 (76.7)	252,203 (76.3)	265,574 (75.5)	15,240 (77.7)
≥65	141,525 (21.1)	129,160 (21.4)	141,206 (21.8)	152,841 (22.5)	8251 (20.1)
Sex					
Female	210,664 (51.2)	203,757 (51.1)	215,102 (51.2)	225,785 (51.3)	12,518 (51.1)
Male	175,784 (48.8)	172,540 (48.9)	186,877 (48.8)	201,138 (48.7)	11,478 (48.9)
Race/ethnicity					
Hispanic	29,442 (16.9)	29,622 (16.8)	31,549 (16.6)	33,509 (17.2)	2472 (23.8)
Non-Hispanic					
Black/African	29,018 (11.8)	28,353 (12.0)	29,424 (11.8)	31,790 (12.1)	1795 (9.7)
White	293,673 (62.6)	282,435 (62.4)	301,206 (62.3)	297,255 (59.7)	17,099 (52.4)
Other people of color^b^	26,127 (8.7)	27,566 (8.8)	30,006 (9.3)	27,901 (10.9)	1861 (14.1)
Household income					
<$25,000	76,116 (20.5)	68,201 (18.9)	51,942 (14.0)	48,237 (12.5)	2852 (12.2)
$25,000–$49,999	75,300 (18.3)	70,782 (17.6)	84,761 (19.9)	80,687 (19.5)	4640 (17.9)
≥$50,000	162,467 (42.4)	163,654 (43.2)	180,481 (44.4)	188,496 (44.6)	11,247 (45.7)
Refused/unsure/don't know	72,565 (18.9)	73,660 (20.3)	84,795 (21.7)	85,507 (23.3)	5257 (24.2)
Physical health (days of not good physical health)					
0–13	326,677 (87.7)	330,061 (90.2)	348,507 (89.2)	363,607 (87.5)	20,228 (87.2)
14–30	52,727 (12.3)	40,041 (9.8)	46,460 (10.8)	55,305 (12.5)	3262 (12.8)
Raising children (≥1)					
Yes	97,093 (34.9)	97,740 (33.9)	101,095 (33.0)	98,517 (31.5)	6052 (33.7)
No	281,981 (62.6)	271,006 (63.6)	291,536 (64.3)	292,054 (64.4)	16,932 (60.8)
Refused	7374 (2.5)	7551 (2.5)	9348 (2.7)	12,355 (4.1)	1012 (5.5)
Anxiety or depression	44,723 (12.9)	43,834 (12.7)	49,050 (13.9)	56,281 (15.0)	3143 (14.5)

a Sample sizes are unweighted, and percentages are weighted to be representative of the US adult population each year.

b Other people of color include non-Hispanic Asian, American Indian/Alaskan Native, Native Hawaiian/Pacific Islander, other races, and multiracial.

We employed slope models to account for slope given by the estimate of trend.^35^ The final intervention models were determined by evaluating the plots of the autocorrelation function and partial autocorrelation function, estimating intervention components, and applying diagnostic tests, including the Bayesian information criterion, Ljung–Box, and Stationary *R*^2^.^31^^,^^33^ The autocorrelation function and partial autocorrelation function plots of residuals (Supplementary Figure S2) were generated to check for significant autocorrelation in the residuals and determine the order of autoregressive and moving average terms. We also plotted the residuals from ARIMA models (Supplementary Figures S3 and S4) to identify any non-random patterns that might suggest model misspecification. The Bayesian information criterion is a model selection criterion that helps evaluate the fit of an ARIMA model, which balances model fit and complexity, with lower Bayesian information criterion values indicating a better-fitting model relative to others. The Ljung–Box Q statistic was used to assess whether the residuals of the model are independently distributed (ie, white noise). This test checks for the presence of autocorrelation in the residuals over different lags. A non-significant result indicates that the residuals do not exhibit significant autocorrelation, suggesting a well-specified model. The Stationary *R*^2^ provides a measure of the proportion of variance explained by the fitted ARIMA model. Additional statistical details of the identification and estimation of ARIMA models are documented in prior publications.^36^^,^^37^

Stratified slope models were constructed for age, sex, race/ethnicity, household income,^38^ physical health,^39^ and whether raising children in the household. It was crucial to include demographic data due to known disparities in mental health across different population groups. As ARIMA procedures account for mean values at all previous time points, we were able to assess whether each COVID-19 vaccine rollout phase was associated with a significant change in the prevalence of anxiety and depression among the total US adult population and subpopulations. We calculated 95% confidence intervals (CIs) for each intervention variable in the ARIMA models. We used complete case analyses to handle missing data when calculating the prevalence of anxiety and depression. ARIMA analyses were performed in SPSS version 25. A 2-tailed p < 0.05 was considered statistically significant.

### Deep Learning

To further validate the impact of phased vaccine rollout on trends in the estimated prevalence of anxiety and depression among the general US adult population, we employed a Long Short-Term Memory (LSTM)-based Recurrent Neural Network. This Deep Learning-based analytical technique facilitates a detailed exploration of temporal dynamics in the trends. Importantly, we aim to compare the findings from the LSTM model with those obtained through traditional statistical approaches (in our case, ARIMA). This comparison might help provide more robust evidence on the impact of phased vaccine rollout and offer timely insights into the potential advantages of employing Deep Learning-based time-series analyses in mental health research.

#### LSTM

When studying changes over time, such as trends in the prevalence of anxiety and depression, LSTN-based Recurrent Neural Network can be a powerful analytic tool. Unlike simpler models that process data in isolation, LSTM networks excel at understanding sequences—like the evolvement of anxiety and depression prevalence over time—because they can remember and integrate information from the past, which is a key advantage when trying to predict trends based on historical data. Traditional time series models might struggle with remembering/utilizing long-term patterns or fail to consider how events from weeks or months ago might influence the present. LSTM networks, however, are built to handle these long-term dependencies. A unique design that manages information with something similar to a memory system allows LSTM to retain important details for as long as needed and discard what is irrelevant. This feature is especially useful in time series analyses, where causes and effects can span long periods.

The core of LSTM's architecture includes components called gates. These gates control the flow of information: deciding what to keep in memory, what to update, and what to output. This mechanism helps LSTMs learn from sequences without getting overwhelmed by the volume of data or forgetting critical information over time. Thus, LSTMs bring the depth of learning necessary to grasp the complexities of time series data, making them a strong research approach to uncover patterns and make predictions in time series analyses. Their ability to learn from and remember extensive sequences of data offers a unique advantage, paving the way for deeper insights into temporal trends. We provided a detailed description of LSTM's architecture in the online Supplementary Methods.

### Interrupted time series model based on LSTM

**Fig. 1:**
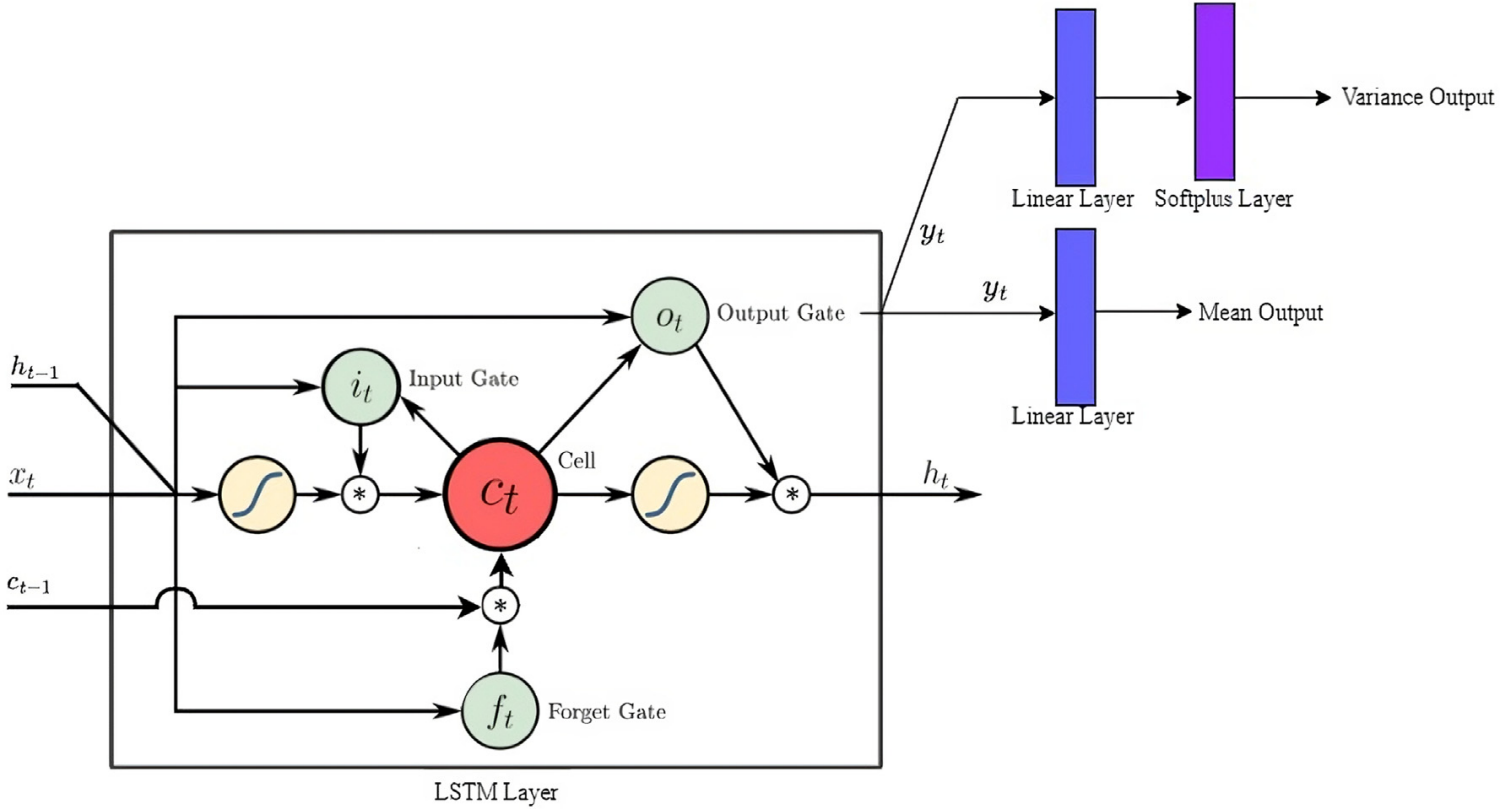
Long short-term memory (LSTM) based Recurrent Neural Network (RNN) Model Architecture.

Fig. 1 shows our model architecture built upon an LSTM-based Recurrent Neural Network, specifically designed for sequence-to-sequence tasks that require intricate handling of temporal dependencies. At the core of our model lies the LSTM layer, crucial for dissecting input sequences to unearth crucial time-related patterns within the data. This feature is particularly vital for tasks where the context of the sequence greatly determines the outcome, such as in time-series forecasting or natural language processing. Central to our investigation is the model's capacity to track the progression of the estimated anxiety and depression prevalence over time, taking into account the significant interruptions of COVID-19 and vaccine rollouts. By analyzing these associations, we aim to provide a detailed understanding of their impacts on trends in anxiety and depression prevalence. Following the LSTM's processing, the model architecture splits into two pathways via linear layers to predict the mean and variance, respectively. To ensure positivity in variance predictions, a Softplus function is applied, which is defined as:

$$Softplus\left(x\right)=\log\left(1+e^{x}\right)$$

Hence, we adapted the LSTM model for sequence modeling and variational inference of the changes in anxiety and depression prevalence over time. It examined the potential impact of key interruptions (ie, vaccine rollout phases) on trends in anxiety and depression.

#### Loss function

We use negative log-likelihood (NLL) loss for a Gaussian distribution. The NLL loss is ideal for training models to estimate the uncertainty of that prediction based on our output μ and σ. NLL can be expressed as:

$$L\left(y,\mu ,\sigma \right)=\frac{1}{2}\log\left(2\pi \sigma^{2}\right)+\frac{{\left(y-\mu \right)}^{2}}{2\sigma^{2}}$$

where *y* is the observed value, μ is the mean of the predicted values, and σ is the standard deviation of the predictions.

#### Input data

Our input data is a time series from 2019 to February 2023, focusing on a single feature referred to as the estimated prevalence of anxiety and depression among the general US adult population. Additional binary features were introduced, representing specific interruptions/interventions within the time series, enabling the model to incorporate the effects of these interventions on the predicted variable.

#### Hyper-parameter setting and training

In the training phase of our model, we leveraged the Adam optimizer for efficient gradient descent. We partitioned our data into a training set and a test set. Within each epoch, we calculated the mean square error (MSE) between the predicted values (μ) and the ground truth for both the training and test sets to ensure a balanced trade-off between underfitting and overfitting while also considering computational efficiency. When trained for 10 epochs, the model exhibited high performance. Each epoch consisted of a complete pass over the entire training dataset, where the model's weights were updated in an effort to minimize the loss function. In the final model, the time window was 12, and the learning rate was 0.001. The machine learning algorithms were implemented using Python 3.9.16 and Pytorch 2.0.1 libraries.

### Role of the funding source

No funding was involved in this study.

## Results

**Fig. 2:**
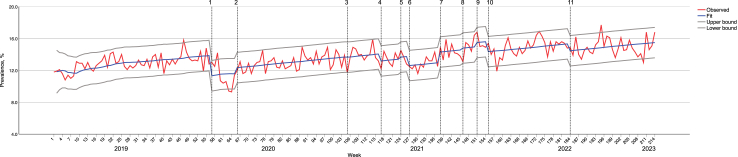
ARIMA multi-intervention interrupted time series analysis for prevalence of anxiety and depression among US adults, Jan 2019 to Feb 2023. **1:** The findings of a novel virus that caused severe unknown pneumonia (later known as COVID-19), first defined as SARS-CoV-2, were published by the CDC and made available to the public. (January 10, 2020). **2:** The Trump Administration declared a nationwide emergency in light of the public health threat due to COVID-19 (March 13, 2020). **3:** Phase 1: healthcare workers and certain at-risk groups were eligible for COVID-19 vaccines (December 11, 2020). **4:** The US Department of Health and Human Services (DHHS) directed all education/childcare workers to be eligible for COVID-19 vaccines, which led to a significant increase in vaccination coverage/rates (March 2, 2021). **5:** Phase 2: all US individuals aged 16+ were eligible for COVID-19 vaccines (April 19, 2021). **6:** Adolescents aged 12–15 were eligible for COVID-19 vaccines (May 10, 2021). **7:** CDC released findings that showed an increase in breakthrough infections of COVID-19, suggesting concerns over the Delta variant (July 30, 2021). **8:** The first COVID-19 booster dose of vaccines was available to certain at-risk US populations (September 22, 2021). **9:** Children aged 5–11 were eligible for COVID-19 vaccines (October 29, 2021). **10:** All US adults were eligible for a booster dose of vaccines (November 21, 2021). **11:** Children aged 6 months to 5 years old were eligible for COVID-19 vaccines (June 18, 2022).

Of 1,615,643 participants (Table 2), 386,448 (238,814 [77.4%] aged 18–64 years, 210,664 [51.2%] females, 293,673 [62.6%] non-Hispanic White) were from 2019, 376,297 (239,469 [76.7%] aged 18–64 years, 203,757 [51.1%] females, 282,435 [62.4%] non-Hispanic White) were from 2020, 401,979 (252,203 [76.3%] aged 18–64 years, 215,102 [51.2%] females, 301,206 [62.3%] non-Hispanic White) were from 2021, 426,923 (265,574 [75.5%] aged 18–64 years, 225,785 [51.3%] females, 297,255 [59.7%] non-Hispanic White) were from 2022, to 23,996 (15,240 [77.7%] aged 18–64 years, 12,518 [51.1%] females, 17,099 [52.4%] non-Hispanic White) were from January–February 2023, representing 236.5, 242.5, 225.6, 255.3, and 17.0 million US adults during each year/time period respectively. Results of diagnostic tests suggested the best fit of the final ARIMA models. In the main model (ARIMA [0,1,1], Stationary *R*^2^ = 0.43) with slope given by the estimate of the trend constant including all participants (Table 3), results revealed an overall upward trend in the prevalence of anxiety and depression from 2019 to February 2023 (Fig. 2). The phase of prioritization of COVID-19 vaccines for educational/childcare workers (interruption **4)** was significantly associated with a 0.93 percentage-point (95% CI −1.81 to −0.04, p = 0.041) reduction in the estimated prevalence. The phase of booster rollout for all US adults (interruption **10**) was significantly associated with a 1.28 percentage-point (95% CI −2.32 to −0.24, p = 0.017) reduction in that estimated prevalence, and the phase of vaccine authorization for children aged 6 months to 5 years old (interruption **11**) was associated with a 0.89 percentage-point (95% CI −1.56 to −0.22, p = 0.010) reduction. In comparison, other rollout phases (interruptions **3**, **5**, **6**, **8**, **9**), such as Phase 1, 2, and authorizations for adolescents, children aged 5–11, and boosters for at-risk populations, were not associated with a significant change in the estimated prevalence.

**Table 3:** Muti-intervention interrupted time series analysis model statistics for the associations of COVID-19 vaccine rollout phases with anxiety and depression among US adults, January 2019–February 2023.

Variable^a^	ARIMA model (p,d,q)^b^	Ljung–Box Q	Stationary *R*^*2*^	Estimate (95% CI)^c^	p value
General US adult population	0,1,1	24.16	0.43		
Constant				0.034 (0.02–0.05)	<0.0001
MA				1 (−0.50 to 2.50)	0.19
COVID-19 Vaccine Rollout Phase					
Phase 1				−0.04 (−0.74 to 0.66)	0.91
Prioritization for educational/childcare workers				**−0.93 (−1.81 to −0.04)**	0.041
Phase 2				−0.70 (−1.94 to 0.53)	0.27
Authorization for adolescents aged 12–15				−1.16 (−2.40 to 0.09)	0.07
First Booster for at-risk populations				0.23 (−0.87 to 1.32)	0.68
Authorization for children aged 5–11				0.51 (−0.77 to 1.79)	0.44
Booster for all US adults				**−1.28 (−2.32 to −0.24)**	0.017
Authorization for children aged 6 months to 5 years old				**−0.89 (−1.56 to −0.22)**	0.010
Pandemic-related event					
Identification of SARS-CoV-2				**−2.55 (−3.34 to −1.77)**	<0.0001
National emergency declaration				0.74 (−0.04 to 1.52)	0.06
CDC updated guildline concerning delta variant				**1.32 (0.42 to 2.21)**	0.004
Age 18–64	0,1,1	27.33	0.45		
Constant				0.04 (0.03–0.06)	<0.0001
MA				1.00 (0.66–1.34)	<0.0001
COVID-19 Vaccine Rollout Phase					
Phase 1				0.43 (−0.43 to 1.30)	0.33
Prioritization for educational/childcare workers				**−1.15 (−2.25 to −0.05)**	0.041
Phase 2				−0.72 (−2.24 to 0.80)	0.36
Authorization for adolescents aged 12–15				−1.33 (−2.86 to 0.20)	0.09
First Booster				0.17 (−1.17 to 1.51)	0.80
Authorization for children aged 5–11				0.60 (−0.98 to 2.17)	0.46
Booster for all US adults				**−1.37 (−2.66 to −0.08)**	0.039
Authorization for children aged 6 months to 5 years old				**−1.52 (−2.33 to −0.70)**	0.0003
Pandemic-related event					
Identification of SARS-CoV-2				**−2.95 (−3.92 to −1.98)**	<0.0001
National emergency declaration				0.48 (−0.48 to 1.44)	0.33
CDC updated guildline concerning delta variant				**1.43 (0.33–2.53)**	0.011
Age ≥65	0,0,0	27.06	0.19		
Constant				7.63 (7.29–7.98)	<0.0001
COVID-19 Vaccine Rollout Phase					
Phase 1				0.22 (−0.63 to 1.08)	0.61
Prioritization for educational/childcare workers				0.05 (−1.01 to 1.11)	0.93
Phase 2				0.37 (−1.18 to 1.92)	0.64
Authorization for adolescents aged 12–15				−1.09 (−2.77 to 0.60)	0.21
First Booster				−0.14 (−1.62 to 1.33)	0.85
Authorization for children aged 5–11				−0.20 (−1.93 to 1.54)	0.83
Booster for all US adults				0.29 (−1.09 to 1.66)	0.69
Authorization for children aged 6 months to 5 years old				**0.79 (0.12–1.46)**	0.021
Pandemic-related event					
Identification of SARS-CoV-2				−0.61 (−1.54 to 0.32)	0.20
National emergency declaration				**0.97 (0.02–1.93)**	0.048
CDC updated guildline concerning delta variant				**1.28 (0.08–2.48)**	0.038
Female	0,1,1	22.06	0.49		
Constant				0.04 (0.03–0.06)	<0.0001
MA				1.00 (0.63–1.37)	<0.0001
COVID-19 Vaccine Rollout Phase					
Phase 1				−0.02 (−1.01 to 0.97)	0.97
Prioritization for educational/childcare workers				**−1.59 (−2.85 to −0.33)**	0.014
Phase 2				−0.69 (−2.45 to 1.06)	0.44
Authorization for adolescents aged 12–15				**−1.81 (−3.54 to −0.08)**	0.041
First Booster				0.01 (−1.52 to 1.54)	0.99
Authorization for children aged 5–11				1.23 (−0.54 to 3.01)	0.18
Booster for all US adults				**−2.03 (−3.47 to −0.58)**	0.006
Authorization for children aged 6 months to 5 years old				**−1.21 (−2.08 to −0.35)**	0.007
Pandemic-related event					
Identification of SARS-CoV-2				**−2.74 (−3.87 to −1.62)**	<0.0001
National emergency declaration				0.97 (−0.09 to 2.03)	0.08
CDC updated guildline concerning delta variant				**1.69 (0.44–2.94)**	0.009
Male	0,1,1	23.60	0.43		
Constant				0.03 (0.01–0.04)	0.0006
MA				1.00 (0.38–1.62)	0.002
COVID-19 Vaccine Rollout Phase					
Phase 1				−0.11 (−1.04 to 0.81)	0.81
Prioritization for educational/childcare workers				−0.34 (−1.51 to 0.84)	0.57
Phase 2				−0.78 (−2.41 to 0.84)	0.35
Authorization for adolescents aged 12–15				−0.49 (−2.12 to 1.14)	0.56
First Booster				0.39 (−1.04 to 1.82)	0.59
Authorization for children aged 5–11				−0.19 (−1.87 to 1.48)	0.82
Booster for all US adults				−0.62 (−1.99 to 0.75)	0.38
Authorization for children aged 6 months to 5 years old				−0.74 (−1.68 to 0.19)	0.12
Pandemic-related event					
Identification of SARS-CoV-2				**−2.44 (−3.47 to −1.41)**	<0.0001
National emergency declaration				0.33 (−0.70 to 1.36)	0.53
CDC updated guildline concerning delta variant				0.98 (−0.19 to 2.14)	0.10
Hispanic	1,0,1	11.8	0.14		
Constant				11.78 (10.93–12.63)	<0.0001
AR				−1.00 (−1.02 to −0.98)	<0.0001
MA				−0.99 (−1.11 to −0.87)	<0.0001
COVID-19 Vaccine Rollout Phase					
Phase 1				0.96 (−1.14 to 3.05)	0.37
Prioritization for educational/childcare workers				−0.96 (−3.58 to 1.65)	0.47
Phase 2				0.59 (−3.23 to 4.40)	0.76
Authorization for adolescents aged 12–15				−2.16 (−6.32 to 1.99)	0.31
First Booster				0.80 (−2.83 to 4.44)	0.67
Authorization for children aged 5–11				0.58 (−3.69 to 4.85)	0.79
Booster for all US adults				0.74 (−2.65 to 4.14)	0.67
Authorization for children aged 6 months to 5 years old				−1.16 (−2.80 to 0.49)	0.17
Pandemic-related event					
Identification of SARS-CoV-2				−0.65 (−2.94 to 1.63)	0.58
National emergency declaration				1.75 (−0.60 to 4.11)	0.15
CDC updated guildline concerning delta variant				1.47 (−1.48 to 4.43)	0.33
Non-Hispanic White	0,0,1	24.90	0.42		
Constant				13.12 (12.73–13.50)	<0.0001
MA				−0.19 (−0.34 to −0.05)	0.010
COVID-19 Vaccine Rollout Phase					
Phase 1				0.17 (−0.77 to 1.12)	0.72
Prioritization for educational/childcare workers				0.51 (−0.67 to 1.68)	0.40
Phase 2				0.67 (−0.99 to 2.32)	0.43
Authorization for adolescents aged 12–15				−1.34 (−3.12 to 0.44)	0.14
First Booster				0.31 (−1.28 to 1.90)	0.70
Authorization for children aged 5–11				0.14 (−1.71 to 1.99)	0.88
Booster for all US adults				−0.74 (−2.23 to 0.75)	0.33
Authorization for children aged 6 months to 5 years old				0.42 (−0.33 to 1.16)	0.27
Pandemic-related event					
Identification of SARS-CoV-2				**−1.65 (−2.68 to −0.63)**	0.002
National emergency declaration				**2.01 (0.95–3.06)**	0.0002
CDC updated guildline concerning delta variant				**2.24 (0.92–3.55)**	0.001
Non-Hispanic Black/African	0,0,0	9.84	0.15		
Constant				13.87 (13.05–14.68)	<0.0001
COVID-19 Vaccine Rollout Phase					
Phase 1				**2.26 (0.24–4.28)**	0.029
Prioritization for educational/childcare workers				−1.20 (−3.70 to 1.31)	0.35
Phase 2				0.72 (−2.95 to 4.38)	0.70
Authorization for adolescents aged 12–15				2.02 (−1.96 to 6.00)	0.32
First Booster				0.70 (−2.78 to 4.19)	0.69
Authorization for children aged 5–11				1.41 (−2.69 to 5.51)	0.50
Booster for all US adults				−1.41 (−4.67 to 1.85)	0.40
Authorization for children aged 6 months to 5 years old				0.26 (−1.32 to 1.84)	0.75
Pandemic-related event					
Identification of SARS-CoV-2				−2.19 (−4.38 to 0.01)	0.05
National emergency declaration				1.1 (−1.16 to 3.36)	0.34
CDC updated guildline concerning delta variant				−0.98 (−3.82 to 1.86)	0.50
Non-Hispanic other people of color	1,0,1	15.26	0.21		
Constant				11.88 (10.94–12.82)	<0.0001
AR				−0.88 (−2.60 to 0.85)	0.32
MA				−0.89 (−2.55 to 0.78)	0.30
COVID-19 Vaccine Rollout Phase					
Phase 1				**2.68 (0.36–5.00)**	0.024
Prioritization for educational/childcare workers				**3.24 (0.35–6.12)**	0.028
Phase 2				−0.26 (−4.48 to 3.95)	0.90
Authorization for adolescents aged 12–15				1.40 (−3.18 to 5.98)	0.55
First Booster				−0.43 (−4.44 to 3.57)	0.83
Authorization for children aged 5–11				3.28 (−1.43 to 7.99)	0.17
Booster for all US adults				−1.20 (−4.95 to 2.54)	0.53
Authorization for children aged 6 months to 5 years old				0.69 (−1.12 to 2.51)	0.45
Pandemic-related event					
Identification of SARS-CoV-2				−2.10 (−4.62 to 0.43)	0.11
National emergency declaration				1.06 (−1.53 to 3.66)	0.42
CDC updated guildline concerning delta variant				1.02 (−2.24 to 4.29)	0.54
Lower household income (<$25,000)	1,0,1	16.37	0.38		
Constant				20.81 (20.10–21.52)	<0.0001
AR				−0.51 (−1.23 to 0.21)	0.17
MA				−0.37 (−1.15 to 0.40)	0.35
COVID-19 Vaccine Rollout Phase					
Phase 1				**3.95 (2.20–5.71)**	<0.0001
Prioritization for educational/childcare workers				1.54 (−0.65 to 3.72)	0.17
Phase 2				3.11 (−0.12 to 6.33)	0.06
Authorization for adolescents aged 12–15				−1.25 (−4.77 to 2.27)	0.49
First Booster				2.10 (−0.96 to 5.16)	0.18
Authorization for children aged 5–11				1.12 (−2.51 to 4.74)	0.55
Booster for all US adults				−0.04 (−2.90 to 2.83)	0.98
Authorization for children aged 6 months to 5 years old				−1.08 (−2.45 to 0.30)	0.13
Pandemic-related event					
Identification of SARS-CoV-2				**−2.96 (−4.87 to −1.05)**	0.003
National emergency declaration				**2.26 (0.29–4.23)**	0.026
CDC updated guildline concerning delta variant				0.34 (−2.14 to 2.82)	0.79
Middle household income ($25,000–$49,999)	1,0,1	14.43	0.42		
Constant				13.88 (13.32–14.43)	<0.0001
AR				−0.97 (−1.06 to −0.88)	<0.0001
MA				−1.00 (−1.88 to −0.12)	0.027
COVID-19 Vaccine Rollout Phase					
Phase 1				1.34 (−0.03 to 2.71)	0.06
Prioritization for educational/childcare workers				0.84 (−0.86 to 2.54)	0.34
Phase 2				1.59 (−0.89 to 4.07)	0.21
Authorization for adolescents aged 12–15				−0.68 (−3.37 to 2.01)	0.62
First Booster				0.21 (−2.16 to 2.58)	0.86
Authorization for children aged 5–11				1.90 (−0.89 to 4.68)	0.18
Booster for all US adults				−1.36 (−3.58 to 0.86)	0.23
Authorization for children aged 6 months to 5 years old				0.46 (−0.62 to 1.53)	0.40
Pandemic-related event					
Identification of SARS-CoV-2				**−1.50 (−2.99 to −0.01)**	0.050
National emergency declaration				**1.90 (0.37 to 3.43)**	0.016
CDC updated guildline concerning delta variant				1.42 (−0.51 to 3.35)	0.15
Higher household income (≥$50,000)	0,1,1	8.36	0.47		
Constant				0.02 (0.01–0.04)	0.008
MA				1.00 (0.34–1.66)	0.003
COVID-19 Vaccine Rollout Phase					
Phase 1				0.06 (−0.91 to 1.02)	0.91
Prioritization for educational/childcare workers				−0.68 (−1.86 to 0.50)	0.26
Phase 2				−1.09 (−2.75 to 0.57)	0.20
Authorization for adolescents aged 12–15				−0.68 (−2.33 to 0.98)	0.42
First Booster				−0.28 (−1.74 to 1.19)	0.71
Authorization for children aged 5–11				−0.21 (−1.90 to 1.49)	0.81
Booster for all US adults				−0.11 (−1.49 to 1.28)	0.88
Authorization for children aged 6 months to 5 years old				−0.53 (−1.53 to 0.47)	0.30
Pandemic-related event					
Identification of SARS-CoV-2				**−1.28 (−2.34 to −0.22)**	0.019
National emergency declaration				**1.26 (0.19–2.32)**	0.021
CDC updated guildline concerning delta variant				**1.51 (0.31–2.70)**	0.014
Self-report good physical health (0–13 days of not good physical health)	0,1,1	16.62	0.44		
Constant				0.02 (0.01–0.04)	<0.0001
MA				1.00 (0.53–1.46)	<0.0001
COVID-19 Vaccine Rollout Phase					
Phase 1				0.15 (−0.50 0.81)	0.65
Prioritization for educational/childcare workers				**−0.91 (−1.74 to −0.08)**	0.033
Phase 2				−0.77 (−1.93 to 0.39)	0.20
Authorization for adolescents aged 12–15				−1.07 (−2.24 to 0.10)	0.07
First Booster				−0.76 (−1.79 to 0.28)	0.15
Authorization for children aged 5–11				0.86 (−0.34 to 2.07)	0.16
Booster for all US adults				−0.97 (−1.95 to 0.01)	0.05
Authorization for children aged 6 months to 5 years old				**−0.87 (−1.55 to −0.18)**	0.014
Pandemic-related event					
Identification of SARS-CoV-2				**−1.75 (−2.49 to −1.01)**	<0.0001
National emergency declaration				**1.36 (0.64–2.08)**	0.0003
CDC updated guildline concerning delta variant				**1.57 (0.72–2.41)**	0.0003
Self-report poor physical health (14–30 days of not good physical health)	0,1,1	16.23	0.45		
Constant				0.04 (−0.01 to 0.09)	0.13
MA				1.00 (0.48–1.52)	0.002
COVID-19 Vaccine Rollout Phase					
Phase 1				−1.82 (−4.93 to 1.30)	0.25
Prioritization for educational/childcare workers				−0.47 (−4.08 to 3.14)	0.80
Phase 2				−0.29 (−5.39 to 4.80)	0.91
Authorization for adolescents aged 12–15				−0.97 (−5.89 to 3.96)	0.70
First Booster				**5.10 (0.81–9.40)**	0.021
Authorization for children aged 5–11				0.59 (−4.46 to 5.63)	0.82
Booster for all US adults				**−4.47 (−8.56 to −0.38)**	0.034
Authorization for children aged 6 months to 5 years old				−0.51 (−2.93 to 1.91)	0.68
Pandemic-related event					
Identification of SARS-CoV-2				**−4.62 (−7.74 to −1.50)**	0.004
National emergency declaration				**3.08 (0.06–6.10)**	0.047
CDC updated guildline concerning delta variant				−1.55 (−5.09 to 1.99)	0.39
Raising children	0,1,1	20.45	0.45		
Constant				0.04 (0.02–0.07)	0.0005
MA				0.99 (0.85–1.14)	<0.0001
COVID-19 Vaccine Rollout Phase					
Phase 1				0.04 (−1.33 to 1.41)	0.95
Prioritization for educational/childcare workers				**−2.18 (−3.88 to −0.47)**	0.013
Phase 2				−0.86 (−3.21 to 1.50)	0.48
Authorization for adolescents aged 12–15				−1.44 (−3.81 to 0.93)	0.24
First Booster				−0.19 (−2.26 to 1.88)	0.86
Authorization for children aged 5–11				0.61 (−1.82 to 3.04)	0.62
Booster for all US adults				−1.01 (−2.99 to 0.96)	0.32
Authorization for children aged 6 months to 5 years old				**−1.42 (−2.59 to −0.25)**	0.018
Pandemic-related event					
Identification of SARS-CoV-2				**−2.08 (−3.62 to −0.54)**	0.009
National emergency declaration				0.16 (−1.30 to 1.62)	0.83
CDC updated guildline concerning delta variant				1.68 (−0.03 to 3.40)	0.056
Not raising children	0,1,1	13.23	0.44		
Constant				0.03 (0.01–0.04)	0.0004
MA				1.00 (−2.81 to 4.81)	0.61
COVID-19 Vaccine Rollout Phase					
Phase 1				−0.05 (−0.96 to 0.85)	0.91
Prioritization for educational/childcare workers				−0.24 (−1.38 to 0.91)	0.69
Phase 2				−0.45 (−2.04 to 1.15)	0.58
Authorization for adolescents aged 12–15				−1.06 (−2.65 to 0.53)	0.19
First Booster				0.45 (−0.95 to 1.85)	0.53
Authorization for children aged 5–11				0.40 (−1.23 to 2.04)	0.63
Booster for all US adults				−1.30 (−2.64 to 0.03)	0.06
Authorization for children aged 6 months to 5 years old				−0.55 (−1.48 to 0.38)	0.25
Pandemic-related event					
Identification of SARS-CoV-2				**−2.58 (−3.60 to −1.55)**	<0.0001
National emergency declaration				1.01 (−0.01 to 2.03)	0.05
CDC updated guildline concerning delta variant				1.11 (−0.03 to 2.25)	0.06

Abbreviation: CI, confidence interval; MA, moving average; AR, autoregressive.

a All intervention variables in the main and stratified models were displayed.

b Diagnostic tests were used to determine the best fit of each ARIMA model (eg, minimum Bayesian Information Criterion, non-significant Ljung–Box Q).

c Values in bold indicate statistical significance of interruptions.

### LSTM

**Fig. 3:**
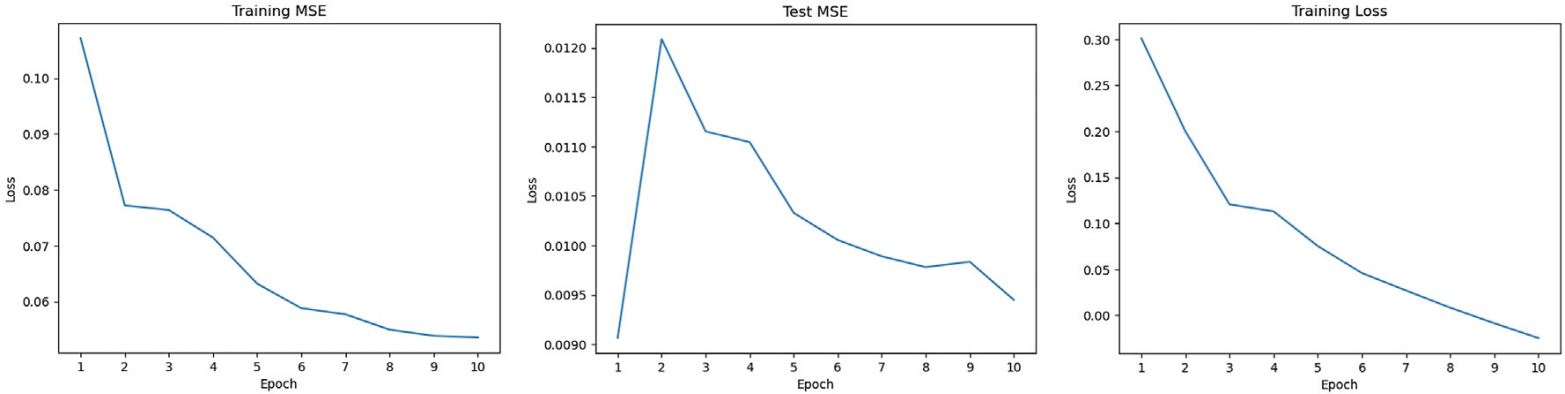
Results of Long Short-Term Memory (LSTM) based Recurrent Neural Network (RNN) Model Training and Validation.

We developed the intervention time series model based on LSTM networks to predict the time series data. The model outputs predictions of both mean and variance, which enable us to compute the 95% CI for these predictions. Training and validation were conducted on the weekly estimated anxiety and depression prevalence among the general US adult population. To find an ideal epoch number, we calculate both MSE on the training and testing dataset (Fig. 3a and b) and the training loss (Fig. 3c). After comprehensive evaluation, we determined that the optimal number of epochs for our model is 10. At epoch 10, the model reached the lowest MSE on the test dataset and had a significant reduction in training loss to a satisfactorily minimal value.

**Fig. 4:**
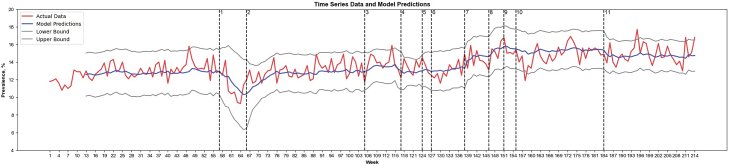
Long Short-Term Memory (LSTM) based Recurrent Neural Network (RNN) Interrupted Time Series Analysis for Prevalence of Anxiety and Depression among US Adults, Jan 2019 to Feb 2023. The red line shows the actual data, capturing the real-time series values observed in the time series. The blue line represents the model's predicted mean; the gray line shows the 95% CI, offering a range within which the true values are expected to fall based on the predicted variance; the black dotted vertical line marks the point of intervention within the time series. **1:** The findings of a novel virus that caused severe unknown pneumonia (later known as COVID-19), first defined as SARS-CoV-2, were published by the CDC and made available to the public. (January 10, 2020). **2:** The Trump Administration declared a nationwide emergency in light of the public health threat due to COVID-19 (March 13, 2020). **3:** Phase 1: healthcare workers and certain at-risk groups were eligible for COVID-19 vaccines (December 11, 2020). **4:** The US Department of Health and Human Services (DHHS) directed all education/childcare workers to be eligible for COVID-19 vaccines, which led to a significant increase in vaccination coverage/rates (March 2, 2021). **5:** Phase 2: all US individuals aged 16+ were eligible for COVID-19 vaccines (April 19, 2021). **6:** Adolescents aged 12–15 were eligible for COVID-19 vaccines (May 10, 2021). **7:** CDC released findings that showed an increase in breakthrough infections of COVID-19, suggesting concerns over the Delta variant (July 30, 2021). **8:** The first COVID-19 booster dose of vaccines was available to certain at-risk US populations (September 22, 2021). **9:** Children aged 5–11 were eligible for COVID-19 vaccines (October 29, 2021). **10:** All US adults were eligible for a booster dose of vaccines (November 21, 2021). **11:** Children aged 6 months to 5 years old were eligible for COVID-19 vaccines (June 18, 2022).

When comparing the predictive performance of ARIMA and LSTM, we found that the LSTM model achieved better performance (Root Mean Square Error [RMSE]: 0.10) compared with the ARIMA model (RMSE: 0.97). The results from LSTM-based multi-intervention time series analysis showed a decrease in anxiety and depression prevalence among general US adults following the phase of prioritization of vaccines for educational/childcare workers (interruption **4**, Fig. 4). We also observed gradual decreases in the estimated prevalence following the phase of booster rollout for all US adults (interruption **10**) and that of vaccine authorization for children aged 6 months to 5 years old (interruption **11**).

### Sensitivity and subgroup analysis

**Fig. 5:**
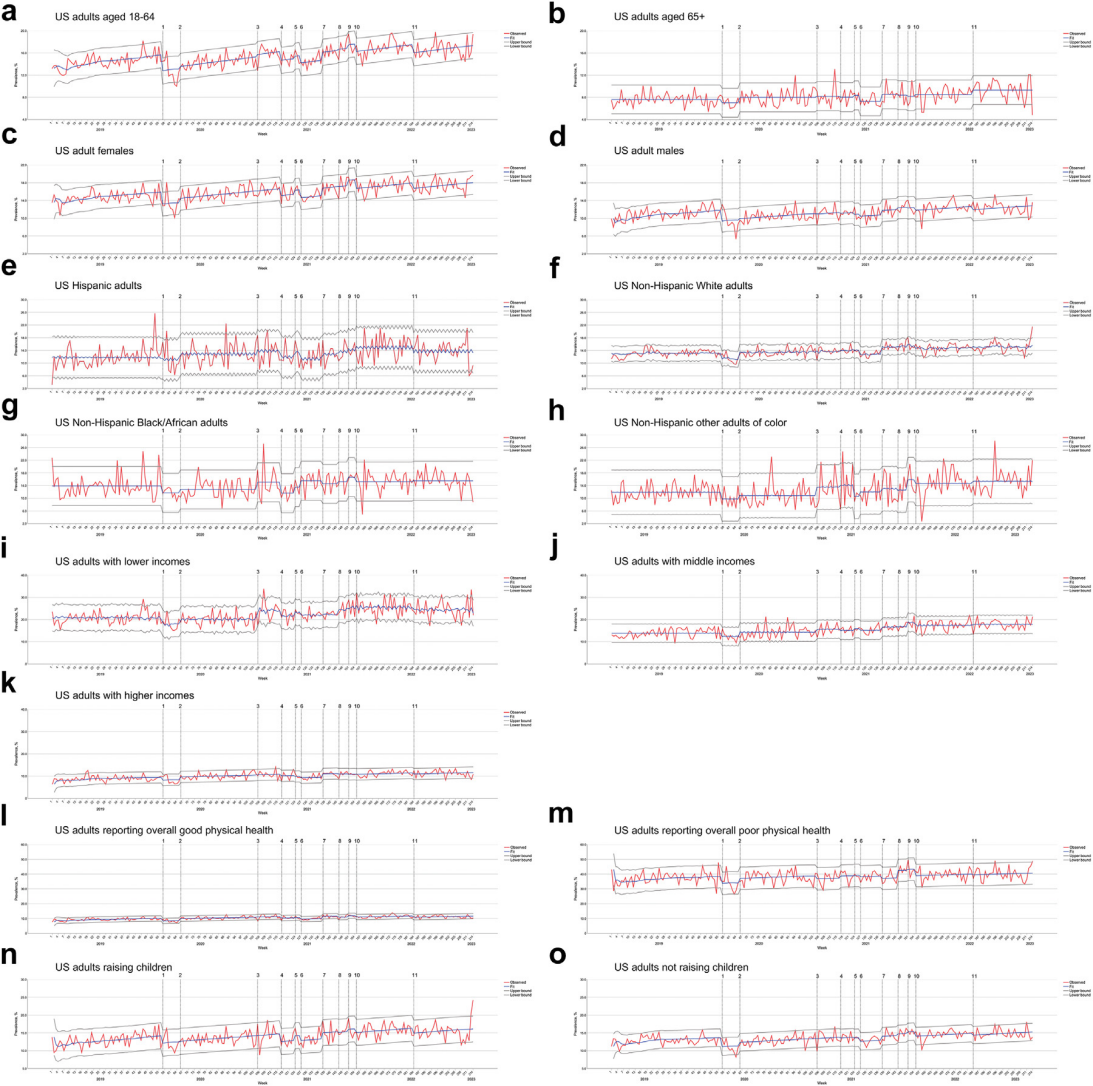
ARIMA Multi-intervention Interrupted Time Series Analysis for Prevalence of Anxiety and Depression by Sociodemographic Characteristics, Jan 2019 to Feb 2023. **1:** The findings of a novel virus that caused severe unknown pneumonia (later known as COVID-19), first defined as SARS-CoV-2, were published by the CDC and made available to the public. (January 10, 2020). **2:** The Trump Administration declared a nationwide emergency in light of the public health threat due to COVID-19 (March 13, 2020). **3:** Phase 1: healthcare workers and certain at-risk groups were eligible for COVID-19 vaccines (December 11, 2020). **4:** The US Department of Health and Human Services (DHHS) directed all education/childcare workers to be eligible for COVID-19 vaccines, which led to a significant increase in vaccination coverage/rates (March 2, 2021). **5:** Phase 2: all US individuals aged 16+ were eligible for COVID-19 vaccines (April 19, 2021). **6:** Adolescents aged 12–15 were eligible for COVID-19 vaccines (May 10, 2021). **7:** CDC released findings that showed an increase in breakthrough infections of COVID-19, suggesting concerns over the Delta variant (July 30, 2021). **8:** The first COVID-19 booster dose of vaccines was available to certain at-risk US populations (September 22, 2021). **9:** Children aged 5–11 were eligible for COVID-19 vaccines (October 29, 2021). **10:** All US adults were eligible for a booster dose of vaccines (November 21, 2021). **11:** Children aged 6 months to 5 years old were eligible for COVID-19 vaccines (June 18, 2022).

As psychological responses to vaccine rollouts and pandemic-related events could have differed across certain population groups, we constructed separate ARIMA models (Table 3), stratified by age, sex, race/ethnicity, household income, physical health, and whether raising children, to investigate potential differential associations (Fig. 5). The phase of prioritization of COVID-19 vaccines for educational/childcare workers (interruption **4**) was significantly associated with a reduction in the estimated prevalence among adults aged 18–64 (−1.15 percentage point, 95% CI −2.25 to −0.05, p = 0.041), females (−1.59 percentage-point, 95% CI −2.85 to −0.33, p = 0.014), individuals raising children (−2.18 percentage-point, 95% CI −3.88 to −0.47, p = 0.013), and those reporting good physical health (−0.91 percentage-point, 95% CI −1.74 to −0.08, p = 0.033). The phase of boosters rollout for all US adults (interruption **10**) was significantly associated with a reduction in anxiety and depression prevalence among adults aged 18–64 (−1.37 percentage point, 95% CI −2.66 to −0.08, p = 0.039), females (−2.03 percentage point, 95% CI −3.47 to −0.58, p = 0.006), and those reporting poor physical health (−4.47 percentage point, 95% CI -8.56 to −0.38, p = 0.034). The phase of vaccine authorization for children aged 6 months to 5 years old (interruption **11**) was significantly associated with a reduction in anxiety and depression prevalence among adults aged 18–64 (−1.52 percentage point, 95% CI −2.33 to −0.70, p = 0.0003), females (−1.21 percentage point, 95% CI −2.08 to −0.35, p = 0.007), individuals raising children (−1.42 percentage-point, 95% CI −2.59 to −0.25, p = 0.018), and those reporting good physical health (−0.87 percentage point, 95% CI −1.55 to −0.18, p = 0.014).

Notably, Phase 1 (interruption **3**) was significantly associated with an increase in anxiety and depression prevalence among Black/African Americans (2.26 percentage-point, 95% CI 0.24–4.28, p = 0.029), other non-Hispanic people of color (2.68 percentage-point, 95% CI 0.36–5.00, p = 0.024), and lower-income individuals (3.95 percentage-point, 95% CI 2.20–5.71, p < 0.0001). The phase of prioritization of COVID-19 vaccines for educational/childcare workers (interruption **4**) was significantly associated with an increased in the estimated prevalence among other non-Hispanic people of color (3.24 percentage-point, 95% CI 0.35–6.12, p = 0.028). The first booster phase was associated with an increase in the estimated prevalence among individuals reporting poor physical health (5.10 percentage-point, 95% CI 0.81–9.40, p = 0.021).

We performed additional sensitivity analyses with different time lag structures for the intervention variables and with an alternative classification of the outcome variable using a different cutoff value (Supplementary Table). The results generally supported the primary findings, including the associations of changes in the estimated prevalence of anxiety and depression with the phase of booster rollout for all US adults and the phase of vaccine authorization for children aged 6 months to 5 years. While the association between the changes in anxiety and depression prevalence and the phase of prioritization of COVID-19 vaccines for educational/childcare workers was not statistically significant, the point estimate was quite compatible with the primary findings.

## Discussion

This current study is the first to investigate the associations between phased COVID-19 vaccine rollout and mental health conditions. Findings from this study provide important information about the potential effects of phased COVID-19 vaccine rollout on population-wide mental health outcomes in US adults. Results of the main ARIMA model showed a modest uptrend in the prevalence of anxiety and depression among US adults from 2019 to February 2023, which corroborates with recent findings indicating a small rise in mental health problems from pre-pandemic to peri-pandemic periods.^27^ Noteworthy is that, despite this upward trend in anxiety and depression prevalence, the results from ARIMA revealed that the estimated prevalence of anxiety and depression dropped following the prioritization for educational/childcare workers on March 2, 2021 (interruption **4**), and the similar results from LSTM model (Fig. 4) lend support to this finding. These findings suggested that this prioritization appeared to alleviate mental health burden among US adults. As many schools (eg, daycare, PreK-12, colleges) had resumed in-person instruction in the fall of 2020, this prioritization might have reduced stress and anxiety among teachers and childcare workers, given their increased risk of exposure and transmission of severe acute respiratory syndrome coronavirus 2 (SARS-CoV-2) due to their close contact with children, adolescents, and caregivers.^40^ Additionally, this prioritization might have had a ripple effect on mental health in the broader community. It is possible that caregivers experienced relief of childcare stress and anxiety concerning the health of their children due to contact with teachers and the continuity of education.^41^^,^^42^ This indirect effect might have contributed to the improved mental health among many US individuals and families, particularly people raising children and adult females who were more likely to be at the forefront of raising and working with children.^43^^,^^44^ Similarly, the vaccine authorization for children aged 6 months to 5 years old (interruption **11**) might have contributed to decreased anxiety/depressive symptoms among many people, including caregivers of young children aged 6 months to 5 years old. Given immature immune systems and the limited resources of viable COVID-19 treatment at that time among young children,^45^^,^^46^ the protection provided by vaccines for this age group might have alleviated anxiety and depressive symptoms among caregivers.

The results from ARIMA and LSTM indicated that the estimated anxiety and depression prevalence decreased after the booster rollout for all US adults on November 21, 2021 (interruption **10**), which might be attributed to the improved vaccine supplies and coverage throughout many communities and regions. In contrast to Phase 1 and 2 during which the country experienced a vaccine shortage, the supply of vaccines during this later booster phase appeared sufficient to meet demand,^47^ and this widespread booster rollout might have contributed to enhanced sense of safety and reduced fear related to Delta and Omicron variants. Further, ongoing health education and outreach initiatives might have also played a crucial role in mitigating vaccine hesitancy and addressing disparities in vaccine access, leading to a lower anxiety and depression prevalence.

However, the results showed that Phase 1 and 2 of COVID-19 vaccine rollout (interruptions **3** and **5**) were not associated with a significant reduction in the prevalence of anxiety and depression among the general US adult population, which might diverge from the expectations that the emergence and availability of vaccines would lead to an overall improvement in mental health.^11^ Given that vaccine distributions were not uniform across the US population due to vaccine shortage, some groups experienced delays in receiving vaccines.^48^ It is possible that this heterogeneity in vaccine distribution led to disparities in access to vaccines, which might have a diluted effect on the overall mental health among US adults. Another possible explanation could be the politicization and persistent misinformation surrounding the safety and efficacy of COVID-19 vaccines during the early stages of the rollout, which might have a mixed influence on mental health across different US populations (eg, pro-vaccine and anti-vaccine groups).^49^^,^^50^

Noteworthy is the finding suggesting an association of Phase 1 vaccine rollout with significant increases in the prevalence of anxiety and depression among Black/African Americans and other non-Hispanic people of color. The recent literature on COVID-19 vaccine hesitancy among Black/African Americans and other people of color reveals that historical and ongoing systemic racism and discrimination across the healthcare systems and society could contribute to vaccine mistrust and distrust.^51^^,^^52^ Research shows that Black/African Americans are less willing to receive the COVID-19 vaccine.^52^, ^53^, ^54^ This hesitancy is largely driven by general mistrust and distrust of healthcare systems, providers, and the US government, rooted in continuous experiences of discrimination, racism, and harmful treatment.^51^^,^^53^^,^^54^ The mistrust and distrust, in turn, might have translated into an unwillingness to accept COVID-19 vaccination.^52^, ^53^, ^54^ Given the heightened risk of COVID-19 complications without vaccination coupled with the stress of systemic racism and healthcare disparities,^8^ it is possible that more Black/African Americans and other non-Hispanic people of color (eg, Asian/Indigenous) experienced mental health problems following Phase 1 vaccine rollout. The finding also suggested that more lower-income individuals experienced anxiety and depression following Phase 1. This might be attributed to a higher proportion of lower-income individuals from Black/African communities,^55^ highlighting socioeconomic inequalities. This said, future research is needed to determine the causal link between vaccine mistrust/distrust and mental health outcomes in Black/African American and other people of color communities.

Further, after the first booster phase, we observed a higher prevalence of anxiety and depression among individuals reporting poor physical health. This increase may be attributed to multiple factors, most notably the challenges associated with vaccine coverage/supply and the reduced effectiveness of the vaccine against the Delta variant.^56^, ^57^, ^58^ Consequently, a greater proportion of people reporting poor physical health might experience increased stress concerning their health and safety. This situation might have, in turn, exacerbated their anxiety and depressive symptoms.

Overall, these findings provide crucial implications for phased disease prevention and intervention strategies in future vaccine administration. Targeted prioritization during phased vaccine rollouts has shown mixed psychological effects across different US populations. Several vaccine rollout phases that encompassed a broad range of groups demonstrated psychological benefits for both the general and specific populations. However, sensitivity and subgroup analyses indicated that highly exclusive phases, limited by vaccine shortages, appeared to adversely affect mental health among marginalized individuals. It is possible that vaccine inequity may contribute to the disparate impacts of phased vaccine rollouts on mental health across US populations. Ensuring sufficient vaccine supply and accessibility could be important for promoting public mental health by enhancing perceptions of public safety and reducing pandemic-related stress and fears. In developing and distributing future vaccines, public health officials and the pharmaceutical industry should consider the role of logistical efficiency and vaccine availability in supporting public mental health. These findings suggested an additional public health benefit of increasing vaccine equity, namely addressing mental health issues.

### Limitations

This study has several limitations. First, the use of retrospective, self-reported survey data may introduce recall bias. Second, although stratified ARIMA models were constructed to detect differential effects of interruptions on the outcome variable in various US population groups, significant effects of interruptions among other population groups categorized by additional sociodemographic characteristics, such as partisanship and religion, may exist. Third, using a single BRFSS question to determine clinically significant anxiety and depression may limit the results despite its good concordance with the PHQ-4, as indicated by previous research.^27^ Namely, the measure's brevity, while advantageous for broad screening, limits its ability to capture the complexity and the full spectrum of anxiety and depressive symptoms. Given that the PHQ-4 and this single BRFSS question screening anxiety and depression do not specifically assess somatic symptoms, the potential confounding effects of physical health conditions on the reported mental health states might lead to over-identification of anxiety and depression among those with high physical comorbidity.

Additionally, BRFSS does not include data on contextual factors (eg, trauma events, psychosocial stressors) linked to mental health outcomes, which does not permit us to consider their effects in our analysis. Thus, although the scale included in BRFSS offers valuable insights into the prevalence of mental health issues at a population level, the results should be interpreted with caution. Large government health surveillance surveys, such as BRFSS, may consider including additional comprehensive screening tools for mental health conditions and questionnaires that account for certain contextual factors to allow for a more comprehensive view and accurate estimation of mental health issues at a populational level.

Moreover, while the BRFSS uses a sophisticated weighting methodology to enhance the representativeness of the sample, it does not include institutionalized populations such as those in nursing homes, prisons, and correctional facilities. This exclusion limits the generalizability of our findings to the entire US adult population. Although the sampling design used by the BRFSS helps minimize the risk of duplicate respondents across years,^59^ there is a small possibility that some individuals might be selected again in different years. The survey does not include unique identifiers, which prevents the assessment of potential duplicate entries. Nonetheless, the robust sampling methodology of the BRFSS, along with its strong reliability and validity demonstrated in previous research,^60^^,^^61^ ensures that findings of the study remain valid despite this limitation. Last, although a smooth function was employed to help mitigate the impact of unmeasured or unobserved confounders, we acknowledge the limitation of being unable to control for all potential confounding factors, which might bias the results. Thus, the results should be interpreted with caution. Future research could benefit from incorporating more granular data, if available, on economic indicators and specific public health policies to strengthen the findings and address this limitation.

### Conclusions

This study suggested that three vaccine rollout phases (ie, prioritization for educational/childcare workers, boosters for all US adults, and authorization for young children aged 6 months to 5 years old) were associated with reductions in anxiety and depression prevalence among the general US adult population. Phase 1 vaccine rollout was associated with increases in anxiety and depression prevalence among Black/African Americans, other non-Hispanic people of color (eg, Asian/Native Americans), and lower-income individuals. Findings of this study should be considered when evaluating and planning initiatives for future phased disease prevention and intervention strategies.

## Contributors

Conceptualization: YZ.

Data curation, verification, and raw data access: YZ, XD, LW.

Formal Analysis: YZ, MF, BG.

Investigation: YZ, MF, BG.

Methodology: YZ, MF, BG, XD, SS.

Project administration: YZ.

Supervision: YZ, BG.

Validation: YZ, MF, BG, XD.

Visualization: YZ, MF.

Writing—original draft: YZ, MF, BG.

Writing—review & editing: YZ, MF, BG, XD, SS, LW.

YZ and XD directly accessed and verified the underlying data reported in the manuscript.

All authors contributed important intellectual content during manuscript drafting or revision and accept accountability for the overall work by ensuring that questions pertaining to the accuracy or integrity of any portion of the work are appropriately investigated and resolved. All authors approved the final manuscript version. All authors have full access to all the data in the study and accept responsibility to submit for publication.

## Data sharing statement

The data that support the findings of this study are openly accessible through the US CDC at: https://www.cdc.gov/brfss/.

## Declaration of interests

We declare no competing interests.
