# Supplementary material for: Impact of phased COVID-19 vaccine rollout on anxiety and depression among US adult population, January 2019–February 2023: a population-based interrupted time series analysis
Source: Lancet Reg Health Am. 2024 Aug 9;37:100852. doi: 10.1016/j.lana.2024.100852 (PMC11519686; doi:10.1016/j.lana.2024.100852)
Supplement: Supplementary Figures and Table [file mmc1.pdf]

Online Supplementary Materials

Impact of phased COVID-19 vaccine rollout on anxiety and depression among US adult population, January 2019 to February 2023: A population-based interrupted time series analysis

Yusen Zhai, PhD[1]*; Mengchen Fan, MS[2]; Baocheng Geng, PhD[2]; Xue Du, PhD[3]; Scott Snyder, PhD[1]; Larrell Wilkinson, PhD[1]

[1] Department of Human Studies, The University of Alabama at Birmingham, Birmingham, AL, USA
[2] Department of Computer Science, The University of Alabama at Birmingham, Birmingham, AL, USA
[3] Heersink School of Medicine, The University of Alabama at Birmingham, Birmingham, AL, USA

Table of Contents

Supplementary Figure 1. Initial Time Series Plot 2

Supplementary Figure 2. ACF and PACF Residual Plots of First Differentiated Time Series 3

Supplementary Figure 3. Model Residual Plot of ARIMA (0,1,1) 4

Supplementary Figure 4. ACF and PACF Residual Plots of ARIMA (0,1,1) 5

Supplementary Methods. Description of LSTM Recurrent Neural Network Architecture 6

ARIMA and LSTM Interrupted Times Series Models 7

Supplementary Table. Sensitivity Analyses of Muti-intervention Interrupted Time Series Analysis Models for the Associations of COVID-19 Vaccine Rollout Phases with Anxiety and Depression among US Adults, January 2019 to February 2023 8

References 9

Supplementary Figure 1. Initial Time Series Plot

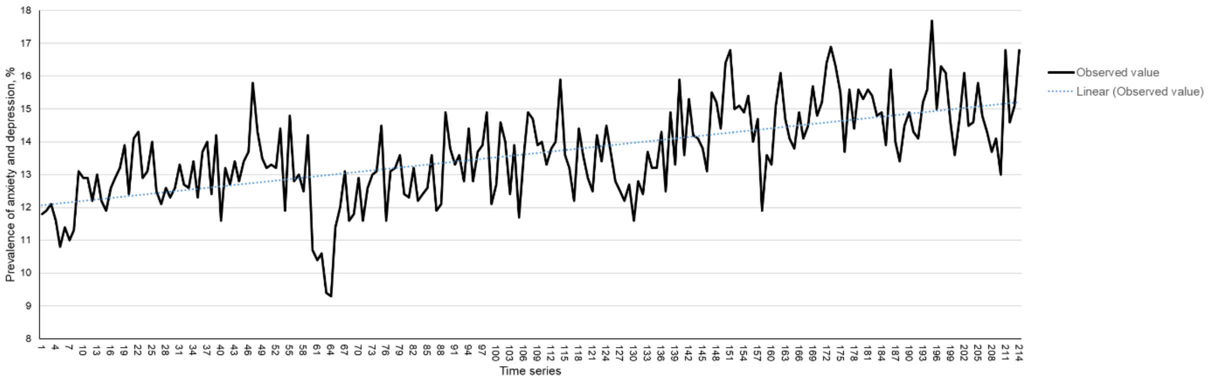

Supplementary Figure 2. ACF and PACF Residual Plots of First Differentiated Time Series

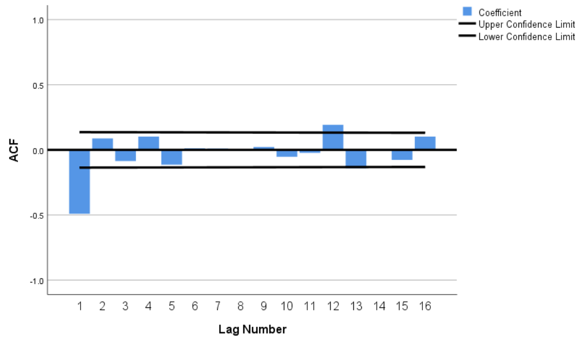

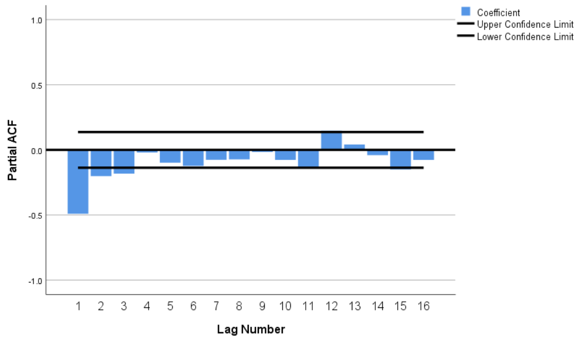

Abbreviation: ACF, autocorrelation function; PACF, partial autocorrelation function.

Supplementary Figure 3. Model Residual Plot of ARIMA (0,1,1)

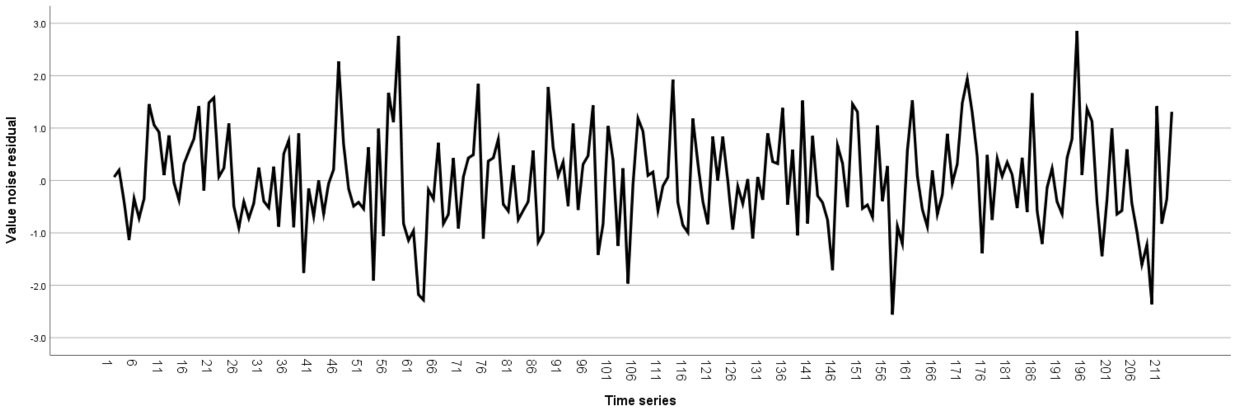

Supplementary Figure 4. ACF and PACF Residual Plots of ARIMA (0,1,1)

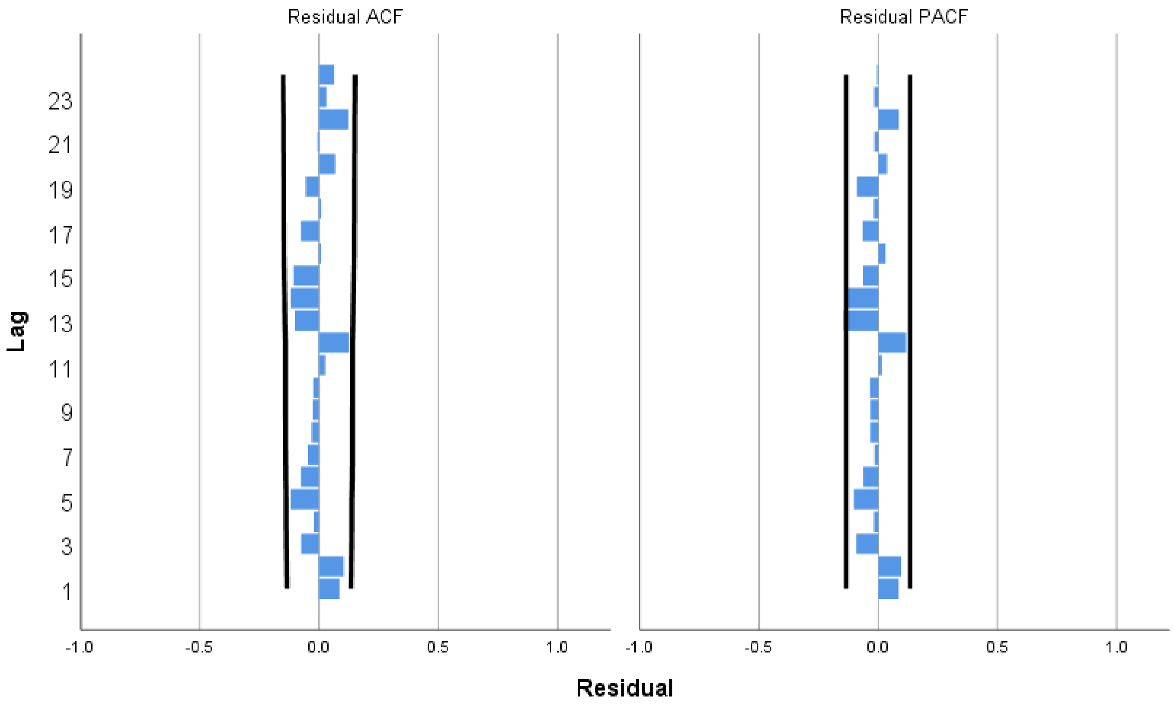

Abbreviation: ACF, autocorrelation function; PACF, partial autocorrelation function.

Supplementary Methods. Description of LSTM Recurrent Neural Network Architecture

Long Short-Term Memory (LSTM)-based Recurrent Neural Network Architecture

Within an LSTM unit, the operations at each time step *t* are governed by a series of equations, which collectively decide how information is processed, stored, and transferred through time. These equations can be understood as follows:

1**. Forget Gate** (f_t): Determines which parts of the cell state are no longer needed and can be discarded, using the equation

$$f_t = \sigma\left(W_f \cdot [h_{t-1}, x_t] + b_f\right)$$

This gate filters out irrelevant past information, ensuring the model focuses only on what is useful.

2. **Input Gate** (i_t): Decides which new information is valuable to keep, calculated by

$$i_t = \sigma(W_i \cdot [h_{t-1}, x_t] + b_i)$$

It identifies valuable current inputs to update the memory state.

3. **Cell State Update**: First, a candidate update ($\widetilde{C_t}$) is created with

$$\widetilde{C_t} = \tanh(W_C \cdot [h_{t-1}, x_t] + b_C)$$

suggesting how to modify the cell state. The actual update to the cell state (C_t) combines this candidate with the old state, influenced by the forget and input gates:

$$C_t = f_t * C_{t-1} + i_t * \widetilde{C_t}$$

This process integrates new insights while discarding outdated information.

4. **Output Gate** (o_t): Determines what part of the cell state will be outputted, using

$$o_t = \sigma(W_o \cdot [h_{t-1}, x_t] + b_o)$$

This gate filters the cell state into a form suitable for making predictions or passing to the next time step.

5. **Hidden State Update** (h_t): The final step,

$$h_t = o_t * \tanh(C_t)$$

produces the hidden state that serves dual purposes: it is both the LSTM's output and the input for the next time step, encapsulating the current understanding of the sequence.

In these equations, σ denotes the sigmoid function, which helps decide whether to let information through a gate. The tanh function produces values between -1 and 1, useful for regulating the information's magnitude. The symbols W and b represent weights and biases, learned parameters that the LSTM adjusts to make accurate predictions. The symbol $*$ represents the Hadamard product (element-wise multiplication) between matrices or vectors. This operation is crucial in LSTM operations where element-wise multiplication is used to apply the gate mechanisms—deciding how much of the previous state to keep, how much of the current input to consider, and how to combine these to update the cell state. The symbol · denotes dot product or matrix multiplication, depending on the context. In the LSTM equations, when referring to the combination of weights (*W*) with the input and previous hidden state (h_{t-1}, x_t), it represents matrix multiplication. This operation combines the input vector and the previous state with the weight matrix to produce a new vector that captures both the new input information and the information carried over from the past. These operations, as described above, manage how information flows through time, making LSTMs capable of remembering and utilizing long-term dependencies in time series data.

ARIMA and LSTM Interrupted Times Series Models

Interpretability

The ARIMA modeling approach is a sophisticated statistical method widely used for time series analysis due to its practicality and interpretability. ARIMA models provide clear parameters (autoregressive [p], integration [d], moving average [q]) that describe the structure of the time series data, making the structure and results of the model (eg, underlying patterns, effects of interventions) more comprehensible for researchers.[1] The coefficients of an ARIMA model have direct interpretations in terms of past values and past errors, which can be easily communicated to a broad audience. LSTM models, as a type of recurrent neural network, are powerful for capturing long-term dependencies and non-linear relationships in time series data.[2] However, the complex architecture of LSTM models often limits their interpretability.[3] LSTMs involve multiple layers and non-linear activation functions, making it difficult to directly interpret the impact of individual input variables or the effect of specific interventions.

Computational Efficiency

ARIMA models involve relatively straightforward calculations and can be quickly estimated using statistical software. Their computational efficiency makes ARIMA models suitable when computational resources are limited.[3] In contrast, LSTM models are computationally intensive due to their complex structure and the need to train multiple layers of neurons.[3] They require more computational power and time, especially for large datasets or when tuning hyperparameters. The training process involves backpropagation through time, which can be resource-intensive.

Non-Linear Relationships

ARIMA models are suited for time series data with linear trends. They struggle to capture non-linear patterns when dealing with complex time series data that exhibit non-linear trends.[2,3] Conversely, LSTM models are capable of modeling non-linear relationships due to their non-linear activation functions and ability to learn from sequential data.[2,3] They can model complex, non-linear patterns and interactions within the data, making them suitable for time series with intricate dependencies and non-linear dynamics.

Supplementary Table. Sensitivity Analyses of Muti-intervention Interrupted Time Series Analysis Models for the Associations of COVID-19 Vaccine Rollout Phases with Anxiety and Depression among US Adults, January 2019 to February 2023

	Model 1 (primary model)			Model 2 (2-week lag)[a]			Model 3 (8-week lag)[b]			Model 4 (cutoff value at 14)[c]		
	RMSE	Estimate (95% CI)[d]	p value	RMSE	Estimate (95% CI)	p value	RMSE	Estimate (95% CI)	p value	RMSE	Estimate (95% CI)	p value
	0.97			1.01			0.98			1.02		
COVID-19 Vaccine Rollout Phase												
Phase 1		-0.04 (-0.74 to 0.66)	0.91		0.06 (-0.67 to 0.78)	0.88		-0.13 (-0.82 to 0.57)	0.72		-0.02 (-0.75 to 0.71)	0.96
Prioritization for educational/childcare workers		**-0.93 (-1.81 to -0.04)**	0.041		-0.91 (-1.85 to 0.04)	0.06		-0.79 (-1.67 to 0.09)	0.08		-0.89 (-1.81 to 0.04)	0.06
Phase 2		-0.70 (-1.94 to 0.53)	0.27		-0.30 (-1.59 to 0.99)	0.65		**-2.13 (-3.36 to -0.90)**	0.0008		-0.78 (-2.08 to 0.51)	0.24
Authorization for adolescents aged 12-15		-1.16 (-2.40 to 0.09)	0.07		**-1.46 (-2.74 to -0.18)**	0.027		1.16 (-0.08 to 2.40)	0.07		-1.16 (-2.45 to 0.13)	0.08
First Booster for at-risk populations		0.23 (-0.87 to 1.32)	0.68		-0.16 (-1.28 to 0.96)	0.78		1.06 (-0.02 to 2.15)	0.06		0.14 (-1.00 to 1.27)	0.82
Authorization for children aged 5-11		0.51 (-0.77 to 1.79)	0.44		1.15 (-0.16 to 2.47)	0.09		**-1.75 (-3.02 to -0.48)**	0.008		0.57 (-0.76 to 1.90)	0.40
Booster for all US adults		**-1.28 (-2.32 to -0.24)**	0.017		**-1.41 (-2.48 to -0.35)**	0.010		0.42 (-0.62 to 1.45)	0.43		**-1.22 (-2.31 to -0.14)**	0.029
Authorization for children aged 6 months to 5 years old		**-0.89 (-1.56 to -0.22)**	0.010		**-1.01 (-1.71 to -0.31)**	0.005		**-0.77 (-1.37 to -0.17)**	0.013		**-0.94 (-1.69 to -0.19)**	0.015
Pandemic-related event												
Identification of SARS-CoV-2		**-2.55 (-3.34 to -1.77)**	< 0.0001		**-1.86 (-2.67 to -1.05)**	<0.0001		**-2.90 (-3.73 to -2.07)**	<0.0001		**-2.54 (-3.36 to -1.72)**	<0.0001
National emergency declaration		0.74 (-0.04 to 1.52)	0.06		-0.21 (-1.02 to 0.61)	0.62		**1.54 (0.78 to 2.29)**	0.0001		0.74 (-0.06 to 1.54)	0.07
CDC updated guideline concerning delta variant		**1.32 (0.42 to 2.21)**	0.004		**1.08 (0.15 to 2.00)**	0.023		0.66 (-0.24 to 1.56)	0.15		**1.42 (0.48 to 2.36)**	0.003

Abbreviations: RMSE, root mean square error; CI, confidence interval.
[a] ARIMA model using 2-week lag structure for each intervention variable.
[b] ARIMA model using 8-week lag structure for each intervention variable.
[c] ARIMA model using a different classification (ie, cutoff at 14) of the outcome variable.
[d] Values in bold indicate statistical significance of interruptions.

References:

1. Schaffer AL, Dobbins TA, Pearson S-A. Interrupted time series analysis using autoregressive integrated moving average (ARIMA) models: a guide for evaluating large-scale health interventions. *BMC Med Res Methodol.* 2021; **21**(1): 58. https://doi.org/10.1186/s12874-021-01235-8.
2. Siami-Namini S, Tavakoli N, Namin AS. A comparison of ARIMA and LSTM in forecasting time series. 2018 17th IEEE international conference on machine learning and applications (ICMLA); 2018: IEEE; 2018. p. 1394-1401. https://doi.org/10.1109/ICMLA.2018.00227.
3. Ai Q, Tian H, Wang H, et al. Comparative Analysis of ARIMA and LSTM Model-Based Anomaly Detection for Unannotated Structural Health Monitoring Data in an Immersed Tunnel. *CMES-Computer Modeling in Engineering & Sciences* 2024;**139**(2):1797-1827. https://doi.org/10.32604/cmes.2023.045251.
